# Extraction of Teeth of Pregnant Women

**Published:** 1882-10

**Authors:** 


### EXTRACTION OF TEETH OF PREGNANT WOMEN.

Among other casual communications, Mr. Henry Sewell brought forward a question as to the advisablity of extracting the teeth of pregnant women. Such patients were constantly applying for relief, but when extraction was proposed—it being evident that the tooth was past saving—one was met with the answer that the patient's doctor did not consider that it would be safe for her to undergo the operation ; so the patient continued to suffer, and her strength was reduced by the pain. His own opinion was that this was a sort of prejudice very much on a par with the idea that it was dangerous or wrong to extract a tooth during the acute stage of alveolar abscess; his practice was, during the early stages of pregnancy, to give gas and extract the tooth. In more advanced cases one must be guided somewhat by circumstances, but even in most of these he believed that extraction did no harm. Even if the patient was weak and nervous, the slight shock of the operation did less harm than the exhaustion produced by long-continued pain.

Messrs. F. Canton and A. Coleman said they were frequently asked this question, and never hesitated to answer it in the affirmative. They preferred to give gas in such cases, and took care to give it thoroughly. They had never seen any harm result from the extraction of teeth under these circumstances.

Mr. George Walliss said he never hesitated to operate when an operation was necessary. It happened on one occasion, in the case of a lady who was very near her time, that the child was born within twelve hours after the extraction ; but she had been in great pain previous to the operation, and she had a much easier labor than she would have had with an aching tooth to add to her other troubles.—*Reports Odontological Society of Great Britain in Med. Times and Gazette.*

